# A narrative synthesis of research evidence for tinnitus-related complaints as reported by patients and their significant others

**DOI:** 10.1186/s12955-018-0888-9

**Published:** 2018-04-11

**Authors:** Deborah Ann Hall, Kathryn Fackrell, Anne Beatrice Li, Rachel Thavayogan, Sandra Smith, Veronica Kennedy, Catarina Tinoco, Evelina D. Rodrigues, Paula Campelo, Tânia D. Martins, Vera Martins Lourenço, Diogo Ribeiro, Haúla F. Haider

**Affiliations:** 1 Otology and hearing group, Division of Clinical Neuroscience, School of Medicine, University of Nottingham, Nottingham, NG7 2RD UK; 2 National Institute for Health Research (NIHR) Nottingham Biomedical Research Centre, Nottingham, NG1 5DU UK; 3 School of Medicine, University of Nottingham, Nottingham, NG7 2RD UK; 4 Department of Audiovestibular Medicine, Halliwell Health and Children’s Centre, Bolton NHS Foundation Trust, Farnworth, UK; 5 ENT Department, Hospital Cuf Infante Santo - Nova Medical School, Travessa do Castro, 3, 1350-070 Lisbon, Portugal; 6 WJCR - William James Center for Research, ISPA-Instituto Universitário, Rua Jardim do Tabaco, n°34, 1149-041 Lisbon, Portugal

**Keywords:** Symptoms, Adults, Otology, Audiology, People important outcomes

## Abstract

**Background:**

There are a large number of assessment tools for tinnitus, with little consensus on what it is important to measure and no preference for a minimum reporting standard. The item content of tinnitus assessment tools should seek to capture relevant impacts of tinnitus on everyday life, but no-one has yet synthesised information about the range of tinnitus complaints. This review is thus the first comprehensive and authoritative collection and synthesis of what adults with tinnitus and their significant others report as problems in their everyday lives caused by tinnitus.

**Methods:**

Electronic searches were conducted in PubMed, Embase, CINAHL, as well as grey literature sources to identify publications from January 1980 to June 2015 in which participants were enrolled because tinnitus was their primary complaint. A manual search of seven relevant journals updated the search to December 2017. Of the 3699 titles identified overall, 84 records (reporting 86 studies) met our inclusion criteria and were taken through to data collection. Coders collated generic and tinnitus-specific complaints reported by people with tinnitus. All relevant data items were then analyzed using an iterative approach to narrative synthesis to form domain groupings representing complaints of tinnitus, which were compared patients and significant others.

**Results:**

From the 86 studies analyzed using data collected from 16,381 patients, 42 discrete complaints were identified spanning physical and psychological health, quality of life and negative attributes of the tinnitus sound. This diversity was not captured by any individual study alone. There was good convergence between complaints collected using open- and closed-format questions, with the exception of general moods and perceptual attributes of tinnitus (location, loudness, pitch and unpleasantness); reported only using closed questions. Just two studies addressed data from the perspective of significant others (*n* = 79), but there was substantial correspondence with the patient framework, especially regarding relationships and social life.

**Conclusions:**

Our findings contribute fundamental new knowledge and a unique resource that enables investigators to appreciate the broad impacts of tinnitus on an individual. Our findings can also be used to guide questions during diagnostic assessment, to evaluate existing tinnitus-specific HR-QoL questionnaires and develop new ones, where necessary.

**Trial Registration:**

PROSPERO registration number: CRD42015020629. Protocol published in BMJ Open. 2016;6e009171.

**Electronic supplementary material:**

The online version of this article (10.1186/s12955-018-0888-9) contains supplementary material, which is available to authorized users.

## Background

Tinnitus is a common condition which is usually described as a buzzing, ringing or hissing sound in the ears or in the head. Prevalence estimates vary from 11.9–30.3% of the population depending on the question asked and the age of the population enrolled [1]. Davis and Rafaie [2] reported that tinnitus is “clinically significant” in about 20% of those who experience it. Data from UK clinical records concerning tinnitus that requires professional healthcare assistance indicates an incidence rate of 5.4 new cases for every 10,000 person-years (95% confidence interval: 5.3 to 5.5) [3].

In the case of tinnitus, both diagnostic assessment and evaluation of treatment-related outcomes rely on self-reports by patients because there are no observable clinical signs or objective tests of tinnitus. However, the range of potential tinnitus-related complaints is potentially extremely broad in scope and, because the tinnitus experience is very individualised and personal, those complaints tend to differ between individuals. To effectively discriminate problems experienced by different patients therefore, it is desirable to ask a comprehensive set of questions that are able to capture this diverse range of possible complaints [4]. This ensures that no important effects of tinnitus which might be important for personalizing individual patient management are missed. There has been no comprehensive collection about what all the possible complaints might be. The commonest problems have been proposed as aspects of quality of life such as psychological or emotional effects, impact on lifestyle, sleep disturbance, auditory and health effects [5], but this is by no means exhaustive. These five examples each describe a discrete dimension or aspect of tinnitus; which we call a “domain”.

For the purposes of assessment, multi-item questionnaires have been developed and these ask questions relevant to numerous tinnitus domains. For the individual who seeks help, all the domains in which they experience problems, not just a limited subset, should to be explored to optimize diagnosis and rehabilitation [4]. Yet, since there has been no comprehensive collection about what all the possible complaints might be, there is little consensus among clinicians and researchers as to preference for a “standard” assessment [6]. Kennedy and colleagues [5] analyzed the item content of five common tinnitus-specific HR-QoL questionnaire instruments used for this purpose (Tinnitus Handicap Inventory THI, Tinnitus Severity Index TSI, Tinnitus Reaction Questionnaire TRQ, Tinnitus Handicap Questionnaire THQ, and the Tinnitus Questionnaire/Tinnitus Effects Questionnaire TQ/TEQ). For each questionnaire item, they classified what domain of tinnitus complaint it considered (see Table 1), using category labels that had previously been reported by three patient-centred studies [7–9]. The authors noted that there was a wide variation across the five questionnaires in the domains of tinnitus complaint that were assessed and in the proportion of items within each domain. These findings raise questions about whether these assessment tools are well-suited to effectively discriminate problems experienced by different patients. Certainly those assessing a limited number of domains could miss important aspects of an individual patient’s difficulties. It is interesting to note that questionnaire developers draw on clinical experience, but tend not to provide adequate information on whether and how they established that the included items and subscales are important to patients (Table 1).

**Table 1:** Stakeholder input and data considerations during development of tinnitus-specific patient-reported questionnaire instruments. This table reported the top six most frequently used in clinical trials of tinnitus interventions; all developed in the English language [see 9]

Questionnaire instrument	Patient input	Professional input	Tinnitus constructs (domains or subscales)
Tinnitus Handicap Inventory [39]	Unclear	Yes	Tinnitus handicap (functional; emotional; catastrophic)
Tinnitus Functional Index [21]	No	Yes	Functional impact of tinnitus (intrusiveness; cognition; emotional; sleep; auditory; relaxation; sense of control; quality of life)
Tinnitus Severity Index [67]	Unclear	Unclear	Negative impact of tinnitus
Tinnitus Reaction Questionnaire [55]	Unclear	Yes	Psychological aspects of tinnitus
Tinnitus Handicap Questionnaire [43]	Yes	Yes	Tinnitus handicap (behavioural, emotional and social; auditory; outlook on tinnitus)
Tinnitus Questionnaire/ Tinnitus Effects Questionnaire [24, 22]	Unclear	Yes	Psychological aspects of tinnitus (intrusiveness; emotional and cognitive distress; sleep; auditory; somatic complaints)

In this article, we therefore fill an important knowledge gap by conducting a comprehensive literature search and narrative synthesis to draw together an in-depth list of patient-reported domains describing different tinnitus-related problems. To date, no-one has yet conducted such a synthesis of the available data, despite the fact that this information is important for understanding the impact of tinnitus on an individual, for guiding patient assessment and for developing new and evaluating existing tinnitus-specific HR-QoL questionnaires.

None of the questionnaire developers listed in Table 1 formally acknowledged a conceptual framework guiding their development work, but it appears to us that tinnitus-specific HR-QoL questionnaire items tend to span the multi-dimensional categories of health captured by the conceptual framework for the World Health Organization (WHO) health-related Quality of Life-100 instrument; namely physical health, psychological state, level of independence, social relations, personal beliefs and their relationship to salient features of their environment [10]. We therefore use this conceptual framework to organize and present the patient-reported domains that we identified from the literature.

Tinnitus affects not only the patient, but also those close to them (typically partners). The experiences of close relatives and friends therefore can provide important insight into the wider impact of tinnitus on everyday life and can be used as an “external barometer” for the needs of the patient. In the case of couples, it can even serve to identify therapeutic needs, to be directed towards either the couple or the spouse alone [11]. While there is a growing body of literature on the impact of tinnitus in those living with the condition; it is unclear what is known about the perspective of significant others.

This review answers the research question concerning what dimensions of tinnitus-related complaints patients and their significant others are reported as being a problem. The main objective of the present review is to collect and synthesise complaints in everyday life that have been reported by people with tinnitus, and also by their significant others. This process generates two perspectives about living with tinnitus: (i) the personal impact of tinnitus from the perspective of the person with tinnitus, and (ii) the personal impact of tinnitus from the perspective of the significant other. Clarifying what complaints are reported by individuals with tinnitus and by significant others would make it easier to identify any potentially important gaps in the content validity of current tinnitus-specific HR-QoL questionnaire instruments. Secondary objectives addressed whether people with tinnitus and their significant others have similar or different perspectives, and whether subtypes of tinnitus and health-related comorbidities influence the nature of the tinnitus complaints that are reported and which countries contributed data to our study findings.

## Methods

*We followed the search strategy, data collection and synthesis methods and the quality assessment as laid out in a predefined protocol* [12]*.* Moreover, to aid later data synthesis, we separately recorded domains identified by open questions from those identified by closed questions (such as Numerical Rating Scales and questionnaires), and we recorded the evidence from closed questions such as if scores were elevated due to tinnitus, compared to controls. It is important to make distinctions between data gathering methods, since open questions best enable patients to have a voice about what is important to them, and not all closed questions necessarily reflect the tinnitus experience as seen from the patient perspective.

### Eligibility criteria

Records were eligible for studies in which adults (≥18 years old) reported tinnitus as a primary complaint, irrespective of whether or not they were attending a clinic for treatment of those complaints, and those reporting data gathered from the significant others of adults with tinnitus. Studies reporting tinnitus as a secondary complaint were excluded. In the context of this review, we used the term “patient” to refer to anyone who has the lived experience of tinnitus. The review included studies reporting data gathered from the significant others of adults with tinnitus.

Records were eligible if tinnitus-related complaints had been collected as part of the screening or baseline assessments, prior to any tinnitus-specific intervention. To be eligible, specific complaints (such as “getting to sleep” and “waking up early”) had to have been collected by the authors and sorted into domains (e.g. “Sleep difficulties”) for reporting, or those complaints constituted items in a subscale or global questionnaire measure. In our review, a domain was defined as a discrete dimension or aspect of tinnitus that can encompass individual complaints with a similar conceptual theme which could be measured by a questionnaire subscale or single-construct questionnaire, but this was not a prerequisite. Collecting and synthesizing data corresponding to individual complaints was not the primary objective of this review and was deemed too great a task for the resources and time available.

Eligible study types were cross-sectional, non-intervention ‘observational’ designs, using techniques such as population surveys, questionnaires, interviews, focus groups and case series. Records were excluded for studies reporting regression modelling predicting tinnitus severity, expert opinions, manufacturers’ articles, practice guidelines, case reports, web-based patient discussion forums and any reviews. If systematic reviews were identified then all included records would be individually assessed for eligibility.

Eligible records were studies published on or after January 1980 conforming to our protocol [12]. To avoid language bias, articles that were not published in English were screened at full text or extracted by native speakers of the written language, using professional colleagues known to the authors.

### Information sources

Information sources were published records which included grey literature, such as conference papers and postgraduate dissertations that had been archived on either Open Grey, PsycEXTRA, DART, ProQuest Dissertations and Theses, Networked Digital Library of Theses and Dissertations, Cos Conference Papers Index (ProQuest) and Web of Science (Thomson Reuters). Grey literature also included website content, searched using Google with the keywords page-by-page up to the point at which a page contains no eligible records. For peer-reviewed articles, electronic databases were searched: PubMed (National Center for Biotechnology Information), Embase (OVID), and Cumulative Index to Nursing and Allied Health Literature (CINAHL, EBSCO). Electronic searches identified publications from January 1980 to June 2015 and were conducted between 12 and 17 June 2015.

Four manual search methods were implemented to increase our confidence in a comprehensive coverage of the available literature and to ensure that all potentially eligible records had been identified. First, on 12 January 2016, we contacted 25 patient associations from across Europe, North America, and Oceania (see Box 2 [12]) to enquire about commissioned reports and unpublished reports relevant to the primary objective. Second, manual searches of reference lists of all articles identified as using open questions (14 March 2016) was conducted. Third, the bibliography of the 71 eligible records was circulated by email (8 July 2016) to 24 tinnitus experts who had previously developed patient-reported instruments (see Box 1 [12]) to identify any candidate records that were missing from our list. Finally, to ensure that the review was up-to-date, manual searches of the top five journals in which eligible records had been sourced (i.e. Ear and Hearing, International Journal of Audiology, Audiology, European Archives of Oto-Rhino-Laryngology and the Journal of Psychosomatic Research), and three additional journals in which eligible records using open questions had been sourced (International Tinnitus Journal, Journal of Speech and Hearing Disorders, and Hearing, Balance and Communication which was formerly the Journal of Audiological Medicine). The final manual search identified publications from June 2015 to December 2017 and was conducted on 20 January 2018.

### Search strategy

The electronic database search strategy required ´tinnitus´ as a title or keyword, in conjunction with additional relevant search terms defined as relevant medical subject headings (MeSH) or text words, wherever possible. The search terms for PubMed and Embase used a combination of terms appearing in the title, keyword or subject, with terms as follows: ´(tinnitus) AND (problem OR complain* OR symptom)´ OR ´(tinnitus) AND (patient OR significant other OR partner OR family)´. For CINAHL and grey literature searches, “tinnitus” was defined as a keyword only. The only exception was ProQuest Dissertations and Theses in which keywords related specifically to co-morbidities, treatments, neural mechanisms and structures were excluded.

### Study selection

Eligibility assessment was independently performed by two co-authors at each key step (i.e. title, abstract, and full-text screening). Discrepancies in title screening were resolved by DAH and HFH, while discrepancies at abstract and full-text stages were resolved by DAH and KF. Those discrepancies in eligibility assessment were predominantly concerned with evaluating the two criteria ‘patient complaints not reported at the domain level’ and ‘factors predicting tinnitus severity’.

### Data collection process

Two coders independently performed duplicate data collection for every study. Overall 13 coders shared the task, predominantly during a 5-day workshop. The number of studies per coder ranged from 5 to 66 (median 9). To minimize observer bias, the workshop included hands-on training with pre-prepared guidance material and electronic data collection form. To promote further data consistency, DAH and KF completed a post-hoc inspection of all 86 studies, collating one data record for each study. This eliminated minor discrepancies in data collection, in particular participant characteristics from the eligibility criteria (Methods) rather than from the reporting of findings (Results). We contacted the corresponding author by email (without reminder) to seek clarification of information, where required.

### Data items

The electronic data collection form included a list of fields relating to eligibility criteria, characteristics of the study population, relevant study findings and other details predefined in the protocol [12]. When information was not reported, the data field recorded ‘not stated’. Tinnitus-related complaints were obtained from the measurement tools used for collecting individual complaints (transcribing the exact interview questions or scales, where relevant, and the responses or scores given). Tinnitus dimensions that included several domains describing patients and significant others’ complaints, were labelled according to descriptions given by the original authors. For studies using closed questions to assess the impact of tinnitus, we did not simply extract data indiscriminately on all subscales or items of the questionnaire. Instead, we extracted data only for those subscales or items that had been highlighted by the study findings or conclusions as reflecting experienced complaints (i.e. those showing elevated scores in people with compared to controls, or demonstrating a substantive treatment-related change over time).

### Synthesis of results

All included records were subjected to a qualitative synthesis that interpreted the data such that new conceptual understanding could emerge [13]. A variety of different terms were used to describe the same underlying theoretical construct, and so we needed to make grouping decisions across the data between studies to cluster together common domain-level concepts across studies. *Before* domain-level grouping of tinnitus-related complaints, t*hree corresponding authors responded to our query by confirming that ‘tinnitus intensity’ was conceptually equivalent to the loudness of the tinnitus percept* [14–16]*.*

Three coders used an iterative approach carefully considering the examples and explanations given by the study authors for each domain of tinnitus-related complaint. The first step required searching for and grouping the domain-level data reported by tinnitus patients under descriptive labels (“codes”) that contained recurring keywords, such as “sleep” and “emotion”. Preliminary domain groupings emerged from the given data taken directly from the full texts (without any abstraction). For the second step, the examples or quotes corresponding to these codes were considered too, and domain-level concepts were reviewed by the same three coders, and re-grouped, where necessary. The same coding scheme was then applied to the domain-level data and examples reported by significant others, in a third step, with new descriptive labels (“codes”) added as required. Steps 2 and 3 involved constantly moving back and forth within the data to identify any overlap or differences in the emerging codes and domain-level groupings. Two new coders then reviewed the classification and made suggestions for revisions based on the domain keywords. Suggestions were shared among the coders, leading to a harmonization of the domain classification [17]. Considering the subjective nature of qualitative analysis, it was agreed that the coding and grouping process was complete once consensus was reached between all coders. The final set of domain labels were carefully reviewed with two lay representatives (native English speakers with tinnitus) to ensure they were understandable by the non-expert.

## Results

### Study selection

**Fig. 1:**
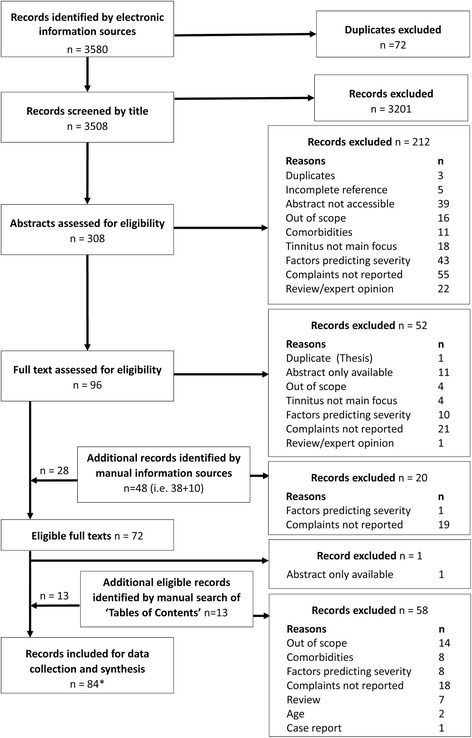
Flow diagram of study records. Eighty-four records yielded 86 independent datasets for synthesis. Note that none of the review articles were systematic reviews

Figure 1 illustrates the flow diagram of all study records (see Additional file 1). Of the 3508 titles identified by electronic searches, 3201 were excluded (‘out of scope’). This left 308 potentially relevant records for the next stage of abstract screening, including a small number of full-texts for those grey literature records. Fourteen records were translated for full-text screening and data collection (see Additional file 2) by trained native speakers. Overall, 212 records were excluded at abstract screening, and 52 records were excluded at the full-text screening and data collection stage with reasons for exclusion given in Fig. 1. Records were excluded for example because references were incomplete, abstracts were not accessible, or full texts were not accessible (see Additional file 3). Records classified as ‘complaints not reported’ all used standard questionnaires and closed questions, or objective quantitative measures; none used qualitative research methods asking open questions.

Manual search methods included contacting patient associations, searching reference lists, asking tinnitus experts for a bibliography list and searching selected journals. Only two associations responded (British Tinnitus Association and Belgian Tinnitus Association) and neither identified any commissioned reports relevant to the primary objective. For the reference list search, four articles were selected from the 96 full-texts assessed for eligibility specifically because they included open questions (see Fig. 1). One was based on authors’ own single open question (Tinnitus Problem Questionnaire) [7], one was based on the authors’ own structured interview [18], and two were based on the Structured Clinical Interview for Personality [19, 20]. From these, 38 potentially relevant records were identified. Three tinnitus experts sent references for potential records, and from these, ten potentially relevant records were identified. The manual search of the seven selected journals identified 13 additional eligible full texts.

The electronic and manual searches created a final list of 84 full-text articles that were included for data collection and data synthesis. References for all of these articles can be found in Additional file 4 and our complete dataset can be found in Additional file 5. Two articles [21, 22] each reported two separate studies, and so this contributed two independent datasets to the synthesis (i.e. 86 studies in total).

### Study characteristics

All 86 included studies assessed the patient experience, whilst two additionally questioned significant others. In total, our review considered data collected from 16,381 patients and from 79 significant others. El Refaie [23] confirmed by email that the number of participating significant others in his study was 57.

The majority of studies used closed questions (e.g. questionnaires, numerical rating scales) as the primary method of collecting individual complaints. Only eight studies asked open questions (885 patients). Open questions were used in the context of structured interviews. Two were based on the American Diagnostic and Statistical Manual of Mental Disorders [24, 25], one on the Psychological Impact of Tinnitus Interview [26], one on the authors’ own structured interview [27], and one on the authors’ own semi-structured interview [28]. The remaining three studies all asked the question from the Tinnitus Problem Questionnaire “Please make a list of the difficulties which you have as a result of your tinnitus. List them in order of importance, starting with the biggest difficulties. Write down as many of them as you can” [7, 8, 29]. None of the included studies collected data using focus groups. Just two studies investigated the perspective of family members; one using a questionnaire about the quality of family life [23] and one using the author’s own questionnaire [30]. Hence, all the data for significant others was collected using closed questions.

### Synthesis of results

#### Complaints relevant to people with tinnitus

**Fig. 2:**
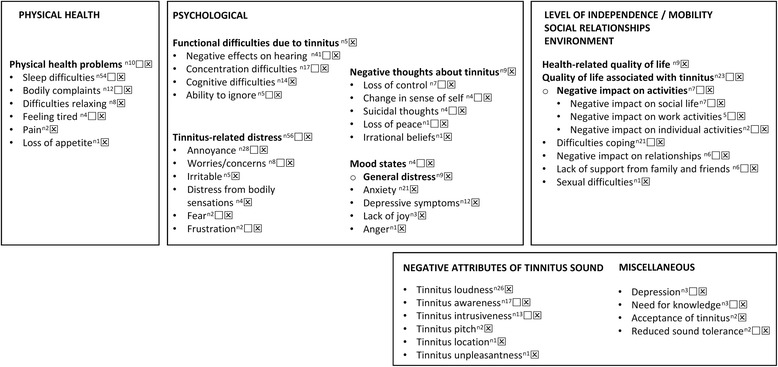
Domain-level grouping created from the responses gathered from patients and significant others. Our domain-level groupings are mapped into the category headings given by the World Health Organization (see headings in capital letters in the top row). Domain-level grouping in the bottom row could not easily be fitted into the WHO nomenclature. Multi-attribute categories are given in bold font; with any denoted with an open bullet point (o) indicating additional multi-attribute domains that have been grouped within the same category. Closed bullet points (●) indicate discrete unidimensional patient-reported domains arising from the data collected. The superscript numbers indicate how many studies in total identified that domain (e.g. ^n11^). An open square (□) denotes that the domain was identified using an open-question format. A crossed square (⊠) denotes that the domain was identified using a closed-question format

Overall 42 discrete unidimensional patient-reported domains were identified by the process of the data synthesis (highlighted by closed bullet points in Fig. 2), with nine multi-dimensional supra-level domains (highlighted in bold font in Fig. 2). A number of descriptors could not be fit into this domain framework because they were broad (e.g. Tinnitus handicap; Tinnitus problem; Tinnitus severity), described multiple theoretical constructs which do not group together (e.g. Effects of tinnitus on the patients social, emotional and physical behaviour), or described external modulators of the tinnitus (e.g. Stressors associated with onset or exacerbation of tinnitus). All of those descriptors that we were unable to allocate to one of the 51 domains are given in Additional file 6.

We have classified the domains into categories inspired by the conceptual framework of quality of life as measured by the WHOQOL-100 [10]. All domains are presented in Fig. 2*, ordered according to the frequency reported across all 86 studies. About two-thirds of domains were identified from both open- (*□*) and closed- (*⊠*) question formats. Any notable exceptions are discussed below. For transparency of reporting,* Additional file 6 gives the domain grouping table that lead to the list of domains reported in Fig. 2. This table contains full details of our chosen domain label and all of the original domain-level terminology used by authors across the source information.

#### Negative attributes of the tinnitus sound

Data synthesis revealed numerous negative attributes of the tinnitus sound. Open-question formats elicited patient reports of ‘*Tinnitus awareness*’ and ‘*Tinnitus intrusiveness*’, also supported by the closed-question data. ‘*Tinnitus loudness’* was a recurring negative attribute, but this always arose from a closed question asked by the investigator (Fig. 2). Occasionally authors’ reporting of loudness was intermixed with annoyance. For example, “subjects tended to report tinnitus that was perceived as moderately loud and annoying” [31]. ‘*Tinnitus pitch’*, *‘Tinnitus location’,* and *‘Tinnitus unpleasantness’* were also identified solely by closed questions (Fig. 2), but these rarely occurred across the 86 studies.

#### Physical health problems

Patients often associated their tinnitus with their physical health. *‘Sleep difficulties’* were the most common physical health difficulty in our dataset; identified using both open- and closed-question formats. Examples indicated difficulties in getting to sleep, in maintaining sleep and in the overall quality of sleep, for example “problems in getting to sleep, waking in the night, waking early” [22], and “tired during the day because tinnitus has disrupted sleep, lie awake at night because of tinnitus, difficult to get back to sleep after waking up at night” [32]. Complaints about physical health included pain, headaches, pressure in ears/head, nausea, dizziness and generally feeling unwell. These were collected and reported as a high-level multi-dimensional construct coded as *‘Physical health problems’* [7, 8, 21, 33]. However, some studies separated out ear/head pain, headaches and muscle tension as symptoms of somatic complaints [34, 35]. We have therefore coded these as a single domain in its own right; coded as *‘Bodily complaints’*. A few studies identified an individual physical symptom such as *‘Feeling tired’* [16], ‘*Pai*n’ [16, 36], and ‘Loss of appetite’ [30].

#### Functional difficulties due to the tinnitus

Five studies clustered a broad range of functional difficulties into a high-level, supra-domain that we have called ‘Functional difficulties due to tinnitus’ [37–41]. This label came from the reporting of the functional subscale of the Tinnitus Handicap Inventory; a closed-question format. Different functional aspects were also coded as separate domains across studies. Most common in this category was *‘Negative effects on hearing’*. This domain included any hearing problem that was attributed by patients to their tinnitus, over and above hearing loss per se. Examples were “difficulties in speech understanding” [42] our translated from Polish, interference with “ability to tell where sounds are coming from” [43], and “distortion of sounds” [28]. Patients also often attributed *‘Concentration difficulties’* to their tinnitus, “being distracted by tinnitus” [44]. Related to the dimension of concentration difficulties was the domain that we have coded as *‘Ability to ignore’* tinnitus, e.g. “Less able to divert their attention from their tinnitus” [27].

A number of studies using closed-question formats also reported *‘Cognitive difficulties’* which encompassed problems with memory and/or attention, such as “Can’t express/tip of tongue, Sudden forgetfulness; Difficulty thinking clearly or remembering” [21, 45]. Five studies grouped a broad range of functional difficulties into a high-level, supra-domain that we have called *‘Functional difficulties due to tinnitus’* [37–41]. This label came from the reporting of the functional subscale of the Tinnitus Handicap Inventory; again a closed-question format.

#### Emotional complaints associated with tinnitus-related distress

Data synthesis revealed many different emotions experienced by patients, but the commonality was that they were all directly attributed to the tinnitus, or some other relevant sensation. Most commonly reported was a high-level construct that we have called *‘Tinnitus-related distress’*. Distress is multi-dimensional in nature and encompasses a constellation of different emotions that other studies had coded as separate domains, such as “Inability to concentrate, Distress/upset, Stress/tension/inability to relax, Irritability, Isolation, Helplessness/frustration” [8] and “[…][…] depression […], anxiety […]” [18], and “loudness, unpleasantness of the noises; […] worries about the persistence of the noises; […] emotional effects (irritability, anger, sadness)” [22]. Annoyance was the most common specific emotion in our dataset. Again these codes predominantly arose from closed questions, but the authors of one of the open question studies [7] pooled “irritation” and “inability to relax” into the same domain as “annoyance” giving some insight into what this construct might mean to patients. A small number of studies did specifically capture, as a separate domain, the sense that tinnitus made them feel *‘Irritable’*. For example, “Tinnitus causes me to feel irritated and angry” [42] translated from Polish. And perhaps related to this construct was also the feeling of *‘Frustration’*: “So ...everyone says ‘you can’t keep on focusing on tinnitus, you must do something nice instead, like travelling’ [...] but I say ‘Yes, but I cannot fly any more” [28]. *‘Worries/concerns’* was another emotion directly associated with the tinnitus experience. The content of the worry appeared to differ from one patient to another, for example “I worry that there is something seriously wrong with my body” [22] and “It becomes a general feeling of worry in me that I did not have before when I did not know so much. It gets back at me, perhaps in many areas. It is how it feels, it sort of spreads in a way” [28]. Two studies reported *‘Fear’*, and so we coded this as a separate emotion because it seemed to capture a stronger sense of emotion. For example, “I’m afraid that the tinnitus will become more disturbing and impairing in the long term” [38].

Finally, our data synthesis identified one negative emotion that was associated with bodily sensations (not with tinnitus per se), which we have termed *‘Distress from bodily sensations’*. This domain was identified by four studies all using a closed-question format. It tended to be presented as a sign of a somatization or somatoform disorder, such as measured by the Tinnitus Questionnaire [14, 44].

#### Negative thoughts about tinnitus

A number of studies identified a construct associated with a *‘Change in sense of self’*. Examples include “self-blame” and a negative “self-image”; “I am retired now, 54 years old and retired, it is not really fun is it, to think that thought?” [28]. Most of these examples arose in response to an open question asked by the investigator. Open questions also identified *‘Loss of peace’*, for example “Peace of mind” [46]. *‘Suicidal thoughts’* was identified from both open and closed questions pertaining to suicide risk [26, 35]. Five studies captured high-level, supra-domain *‘Negative thoughts about tinnitus’*, by asking closed questions. The sense of being unable to “escape tinnitus” and no longer coping with the tinnitus [37, 39], both questions that form part of the Catastrophic subscale of the Tinnitus Handicap Inventory. Similarly, the construct termed *‘Loss of control’* forms a distinct domain of the Tinnitus Functional Index e.g. [21]. *‘Irrational beliefs’* were also identified by a closed question in one study [47].

#### General mood states

In contrast to emotions, we have classified moods as general feelings that are not triggered by tinnitus, nor by any other sensation. Mood states tended not to be reported by patients using open questions, but rather in response to specific questionnaire items. A small number of studies used *‘Mood states’* as a high-level ‘supra-domain’ term, often measured using a generic mood questionnaire [26, 28, 30, 48]. Others used the term *‘General distress’*, a construct that is synonymous with ‘stress’ and was typically measured using a generic stress questionnaire e.g. [48–50]. From our dataset, a recurring specific mood state was *‘Anxiety’*. *‘Depressive symptoms’* was another. Typically, anxiety and depression were measured using a relevant (sub)scale of a closed-item questionnaire, such as the State Trait Anxiety Inventory [15, 51], Beck Depression Inventory e.g. [15, 52], or the Hospital Anxiety and Depression Scale e.g. [16]. Two other mood states relating to feelings not necessarily directed towards the tinnitus were *‘Lack of joy’* (e.g. “frequency of loss of joy”, 44) and *‘Anger’* [48].

#### Health-related quality of life

Many study findings attributed a reduction in quality of life specifically to the tinnitus (i.e. *‘Quality of Life associated with tinnitus*’) [43]. This domain was identified using questionnaire items or subscales [Tinnitus Handicap Questionnaire, 21; Tinnitus Functional Index, 27], and also in response to an open question asked by the investigator [27, e.g. “Impairments in quality of life, reduced stress tolerance, psychosocial withdrawal”). A number of studies defined *‘health-related Quality of Life’* as a generic domain relevant to people with tinnitus but not specifically attributed to the tinnitus. This was most commonly measured using a single-item Numerical Rating Scale e.g. [44, 53].

*‘Negative impact on activities’* was another multi-dimensional domain that emerged from the data synthesis of both open- and closed-question formats. The type of activities were either unspecified “avoided otherwise enjoyable activities, more restricted in their activities” [27], or encompassed several different types of activity in the same author code, such as “interfered with work, less interested in going out” [54]. Within this supra-domain there were three unidimensional domains associated with specific categories of activity; namely social, work, and individual. *‘Negative impact on social activities’* was illustrated by examples such as “General interference with leisure; Less interested in going out; Social life was limited” [21, 28]. *‘Negative impact on work activities’* was exemplified by this description “Work as a situation in which tinnitus had a severe negative impact. Some had stopped working altogether, and others had reduced their working hours or changed workplace/work assignments” [28]. With respect to *‘Negative impact on individual activities’*, an illustrative example was “ability to concentrate, listen to music or read newspapers” [55]. Patients also reported issues relating to relationships with family and friends, for example “I become, yes, some kind of obstacle for, for some things my wife and I might have planned to do together” [28], and these were classified by the category *‘Negative impact on relationships’*.

While some studies mentioned the use of coping strategies without giving examples, our data synthesis did indicate a diversity of cognitive and behavioural approaches to coping in use by patients, and which we have labelled as the domain *‘Difficulties coping’*. Examples spanned avoidance (“I avoid noise due to tinnitus; I avoid silence due to tinnitus; Due to my tinnitus I avoid sporting activities” [38, 46]), using hearing protection (“Due to my tinnitus I try to protect my ears whenever it is possible” [38]), and what the authors called ‘saving face’ (“what is shown to other people” [28]). None seemed specific enough to separate into distinct domains with the exception of *‘Lack of support from family and friends’* (e.g. “I notice people have a hard time trying to understand” [28] and “Family gets aggravated with me” [56]). A final domain in this category was *‘Sexual difficulties’*. There was just one instance of this, and it was assessed in a study using the Chronic Illness Problem Inventory [24].

#### Miscellaneous domains

A small number of other domains were recorded, but could not easily be classified according to the above categories. Of note, a few studies included an assessment that indicated a clinical diagnosis of depression (e.g. Harrop-Griffiths [24] assessed patients against the American Diagnostic and Statistical Manual (DSM III) criteria). No studies reported on a formal clinical diagnosis of anxiety. Other domains were *‘Need for knowledge’* which was exemplified by: “Explaining tinnitus to others” [7], and ‘*Acceptance of tinnitus’* which included the example “Tolerance” [57]. These two domains were identified by few studies.

Also not easily classified in the WHOQOL-100 conceptual framework were two studies reporting a reduced sound tolerance, possibly associated with hyperacusis [28, 58].

### Secondary analyses

Secondary objectives addressed whether people with tinnitus and their significant others have similar or different perspectives, whether subtypes of tinnitus and health-related comorbidities influence the nature of the tinnitus complaints that are reported, and which countries contributed data to our study findings.

#### Complaints reported by *significant others*

Only two studies addressed domains of tinnitus-related complaints reported by 79 significant others in terms of their own personal experience [23, 30]. These questionnaire studies identified ten domains which were all contained within the classification for people with tinnitus (see Additional file 7): *‘Sleep difficulties’*, *‘Negative effects on hearing’*, *‘Mood states’*, and *‘General distress’*, *‘Negative impact on relationships’*, *‘Negative impact on activities’*, *‘Negative impact on social life’*, and *‘Difficulties coping’*, *‘Physical health problems’*, and *‘Need for knowledge’*. *‘Negative impact on relationships’* was the only domain to be identified in both studies.

In addition to comparing the domains identified by patients and significant others, we had planned to conduct two further secondary analyses. The first was to explore whether different tinnitus subtypes might influence different patterns of reported domains. Pre-specified classifications were tinnitus duration (acute versus chronic), tinnitus presence (intermittent versus constant), tinnitus pulsatility (non-pulsatile versus pulsatile), tinnitus severity (mild versus moderate versus severe), co-morbid anxiety, depression and severity of hearing loss. Adequate analysis of each research required a sufficient number of studies either to have enrolled only participants according to individual subtypes, or to have separately characterized and reported complaints according to subtype. Unfortunately, none of the included studies did this for any of the pre-specified classifications. The second was to explore whether a health-related comorbidity might influence different patterns of reported domains. Seventy-one of the studies reported no co-morbidity related inclusion criteria, 10 reported hearing loss, one insomnia, and one hyperacusis. Although this is insufficient to draw any strong conclusions, the form of the co-morbidity was associated with the reporting of the associated complaint. Notably, the study recruiting people with tinnitus and insomnia was one of the studies identifying the ‘*Sleep difficulties’* domain [59], and the study recruiting people with tinnitus and hyperacusis was one of the studies identifying the ‘*Reduced sound tolerance*’ domain [58] (Fig. 2). In addition, three of the 10 studies actively recruiting people with hearing loss identified ‘*Negative effects on hearing*’. [47, 50, 60].

**Fig. 3:**
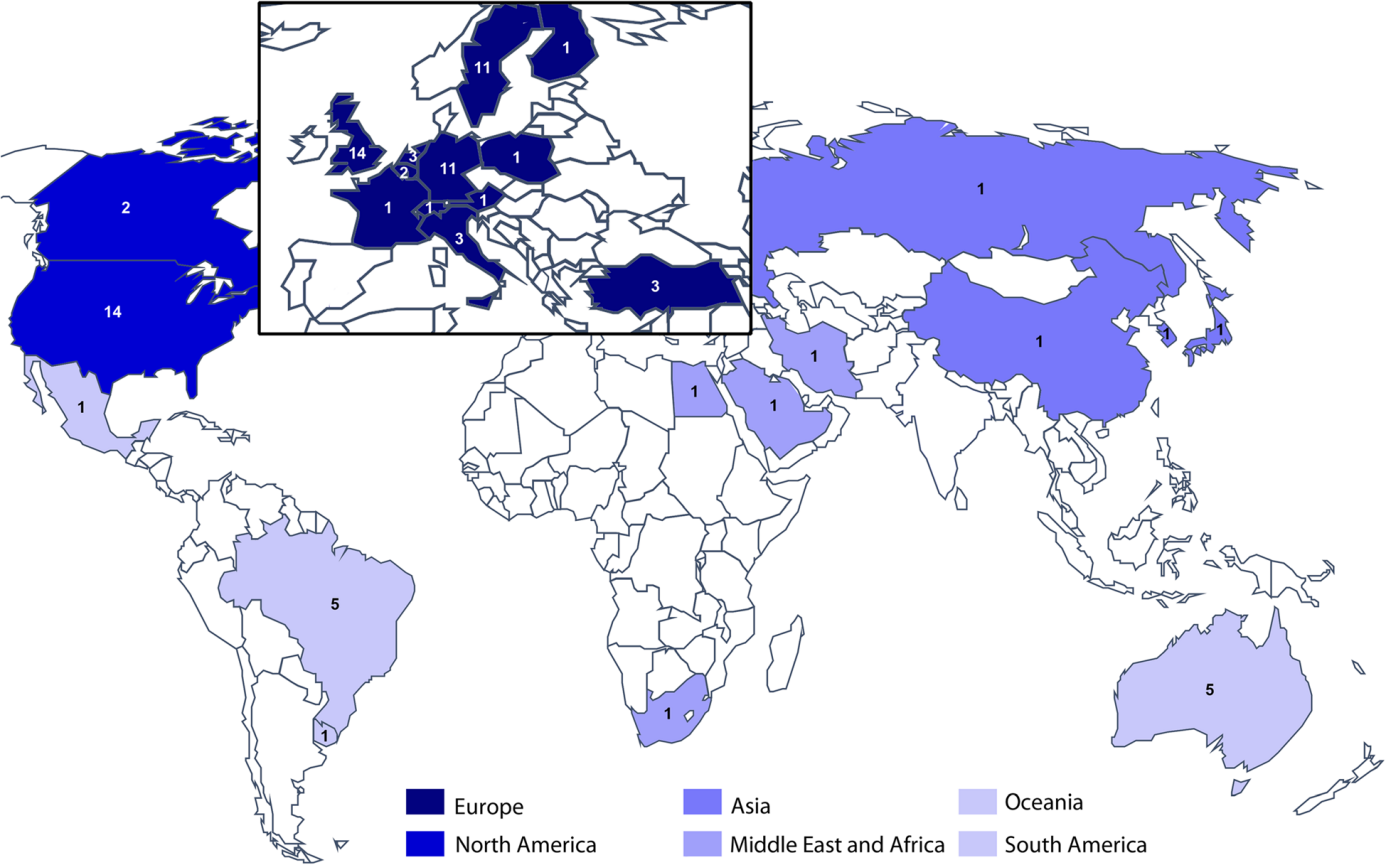
World map illustrating the distribution of study sites for all included studies, inspired by the World Health Organization (WHO) regional classification, but with Region of the Americas separated into North and South and with Australia and separated from Western Pacific region, because of cultural and language differences. Regions are colour coded in different shades of blue and the values denote the number of studies contributing to the review from that country

The final secondary analysis explored which countries contributed data to our findings. The complete dataset included the data item for the country where the study was conducted. Overall, the data predominantly came from UK, USA, Germany and Sweden (shown in Fig. 3), including the two studies investigating significant others (UK) and the four qualitative research studies reported below in Table 2 (UK, Sweden). This observed geographical bias was unlikely to be explained by our study design since we translated all eligible records.

**Table 2:** CASP checklist for records that passed the first two screening questions. ✓ = checklist criterion was met, ✗ = not met, and? = can’t tell

	Research design	Recruitment strategy	Data collection	Relationship/bias	Ethical issues	Data analysis	Statement of findings	Value of the research
Tyler & Baker 1983 [7]	?	?	✓	✗	✗	✗	✓	✓
Sanchez & Stephens 1997 [8]	✓	✓	?	✗	✗	✗	✓	✓
Sanchez & Stephens 2000 [29]	✓	?	?	✗	✗	✗	✓	?
Andersson & Edvinsson 2008 [28]	?	✓	✓	?	✗	✗	✓	✓

### Quality assessment

The protocol described three assessments of the quality of collecting, defining and reporting the domains of tinnitus-related complaints. We evaluated the extent to which investigators used an open questioning format, and then assessed quality for those studies using a qualitative research design. Qualitative research is valuable because it can illuminate the personal meaning of tinnitus without constraining findings by any investigators’ preconceptions and can enable an in-depth exploration or relevant issues. Eight articles used open question interviewing, but four either used a closed format response scale [26, 27] or used patient responses only to make a psychiatric diagnosis [24, 25]. O*nly four remaining records* [7, 8, 28, 29] met the Critical Appraisal Skills Programme (CASP) [61] checklist screening questions confirming that they were qualitative research studies (610 patients in total). Two authors (KH and DAH) then subjected these records to a quality assessment using the remaining eight CASP checklist questions and agreed ratings are given in Table 2. While reporting of findings was adequately detailed, there was no confirmation of ethical approval, no consideration about whether or how data collection might have been affected by the investigator-patient relationship and incomplete reporting of how the text-based data had been analyzed to identify tinnitus domains.

The remaining 80 records (82 independent studies) were subjected to the quality assessment described in the protocol [12], relevant to the degree to which reported findings reflected the heterogeneity of a ‘typical’ tinnitus population. Records were assessed for: (i) justification of sample size, ii) reporting a wide variety of ages (mean and SD), (iii) gender balance (men and women), and (iv) no eligibility criteria that would exclude particular tinnitus subgroups. Each criterion was scored 0, 1 or 2 to give a composite score out of 8. The mean quality score was 5.24 (SD = 1.37). Most poorly handled was the justification for sample size, with 69 studies not given any explanation for why the numbers recruited were sufficient to address the primary question.

## Discussion

The effects of tinnitus on the person with the condition and on their significant others are pervasive and affect the quality of life for all involved. This comprehensive review is important because it has collated and synthesized, for the first time, everyday life tinnitus-related complaints that have been reported by people who have the lived experience of tinnitus and their significant others.

### Comparison with other studies

To our knowledge, no other study has achieved a comprehensive qualitative synthesis of patient-reported complaints associated with tinnitus. Perhaps two of the closest are a systematic review of clinical trials of tinnitus in adults [6], and a qualitative content analysis of tinnitus problems and effects on everyday life according to the International Classification of Functioning, Disability and Health [62]. The findings of these two studies are consistent because they map very well onto concepts that are equivalent to the domains that have emerged from our qualitative data synthesis, albeit sometimes with a slightly different choice of wording (e.g. *‘Tinnitus-related cognitions’* [6] became *‘Negative thoughts about tinnitus’* and *‘Sustaining attention’* [62] became *‘Concentration difficulties’*). The main reason for these differences in wording can be attributed to the novel influence of involving lay people with tinnitus whose role has been to challenge us to find domain labels that were as jargon free as possible. Members of the public were not explicitly involved in our review team, but did influence our choice of domain labels at the reporting stage because of their involvement in an ongoing study as part of the next step in our research programme [63].

Despite our rather strict definition of a domain, our synthesis identified a large number of discrete unidimensional constructs associated with tinnitus complaints. This lengthy classification list differs markedly from all previous studies, but perhaps surprisingly even for those published studies which surveyed tinnitus patients using an open question and then analyzed the resulting patient narratives. For example, Tyler and Baker’s landmark study [7] of 72 patients reported only four domains (hearing, lifestyle, general health, emotional problems). The examples that they gave for the hearing domain map well onto our domain *‘Negative effects on hearing’*, and so do those for health (see *‘Physical health problems’*). However, lifestyle and emotional problems do not, perhaps because Tyler and Baker [7] intermingled a range of different concepts. For example, the examples that they gave for lifestyle we have coded under numerous discrete domains (Sleep difficulties; Tinnitus awareness; Difficulties coping; Negative impact on social life; Negative impact on relationships; Negative impact on work activities; Negative impact on individual activities; Need for knowledge). Indeed, Sanchez and Stephens [8, 29] seem to have also recognized a difficulty with multi-dimensional constructs because in their analysis of data collected using the same procedure as Tyler and Baker [7] they created the additional domain *‘Sleep’*. We consider there to be scientific value in reporting patient-related complaints at the level of individual, discrete health-related constructs, not high-level broad categories. In our experience, both patients and healthcare professionals find these both understandable and highly relevant to their own personal experiences [63].

### Limitations of the study

We acknowledge a potentially limiting factor is that our search identified *only four* qualitative research studies assessing 610 patients [7, 8, 28, 29], and no new qualitative studies were identified in the manual search update but see [62]. Geographical bias was avoided since no records were excluded because of an inability to adequately translate into English.

Given the relative paucity of qualitative methodology to understand tinnitus complaints, as experienced by the patient and their significant others, it is possible that additional complaints might emerge from new research. For example, we are aware of one unpublished study, presented at a recent conference [64], in which the authors collected tinnitus-related complaints from 988 patients using a single open question: “Why is tinnitus a problem?”. However, this new study does not add any new information to the domain-level grouping represented in Fig. 2.

### Future directions

Our findings highlight a number of knowledge gaps each of which be a promising future direction for research. First, the tinnitus-related complaints spanned aspects of physical health, psychological health (i.e. functional, cognitive and emotional), independent activities, social relations, and leisure activities. For the majority of these, we found converging evidence for their relevance to people with tinnitus, through responses to open-format questions as well as group differences in scores on closed-format questionnaires. Although patients typically attributed direct causality to the tinnitus, we noted that these domains are also generic components of well-being that are represented within the WHO conceptual framework of quality of life [10]. This raises an important unanswered question about whether or not a profile of the impact of tinnitus could adequately be measured by a standardised, generic quality of life instrument.

Second, we observed that a small number of patient-reported domains were identified only by directed, closed questions asked by the investigator and were never ‘spontaneously’ reported by patients in response to an undirected, open question. Notably, these included general moods (not triggered by tinnitus) and also the four major perceptual attributes of the tinnitus sound (i.e. its location, loudness, pitch and unpleasantness). These domains highlight discrepancies between the perspectives of patients and healthcare professionals; while they appear to be valued by clinical practitioners, this is not true for patients. This finding also raises a specific dilemma because loudness is a common primary outcome measure in clinical trials [6], and yet it may not be so relevant to patients. Importantly, this review raises concerns about whether tinnitus loudness has sufficient content validity to be an essential item for inclusion, certainly as part of a patient-reported primary outcome instrument when determining the clinical efficacy of an intervention. Again, further research is warranted.

Third, the impact of tinnitus on the patient’s significant other may provide clues on how a couple or family deal with tinnitus in their daily routine and considering such challenges may contribute a more complete clinical profile of a patient undergoing clinical assessment and management. Our review highlights a gap in our knowledge concerning third-party disability because there is a paucity of literature about the effect of tinnitus on significant others. Like hearing loss, many fewer studies are directed at investigating the impact of the condition on close friends and family than on patients themselves. For hearing loss, a recent review identified 24 articles reporting the impact of tinnitus on significant others [65]. However, in the case of tinnitus, third-party disability does not appear to be a topic of growing research interest because we identified only two articles, with the most recent having been published over 10 years ago [23]. This lack of data means that our findings are unlikely to capture all domains relevant to this stakeholder group, and so further research is warranted.

Finally, our findings make a specific contribution to the ambitious roadmap for developing a Core Outcome Set for tinnitus which would set minimum standards for collecting and reporting outcomes in all clinical trials of tinnitus [63]. This review identifies all those patient-reported domains that could be candidates for a Core Outcome Set, thus giving credence to the patients’ viewpoint.

## Conclusions

There is a recognition that measurement instruments used for clinical diagnosis and for evaluation of the outcome of tinnitus interventions should have good content validity (i.e. that their content is an adequate reflection of complaints that are relevant to tinnitus) [66]. The findings of this comprehensive review therefore contribute fundamental new knowledge and a unique resource that will enable investigators to evaluate the relevance to patients of any multi-item patient-reported questionnaire for tinnitus. Clarifying the types of tinnitus-related complaints that are often reported enhances our understanding of the lived experience of patients and highlights important gaps in content validity of current tinnitus-specific HR-QoL questionnaire instruments.

## Additional files

Additional file 1: Table summarising the electronic information sources used to identify the 3580 records. For a description of the abbreviations, see text. (DOCX 17 kb)

Additional file 2: Fourteen non-English language records that were screened and either excluded or included at the full-text stage by native language speakers. ‘Complaints not reported’ = authors reported the global tinnitus score calculated from a multi-attribute questionnaire without reporting the component domains or subscales. (DOCX 18 kb)

Additional file 3: Records that were excluded because either the abstracts and/or full-texts were not accessible, or there was an incomplete reference which meant that the article could not be traced. (DOCX 35 kb)

Additional file 4: References for all 84 full-texts included for data collection and synthesis. (DOCX 22 kb)

Additional file 5: Complete dataset, including our domain coding and quality assessment. (XLSX 520 kb)

Additional file 6: Grouping table reporting the different terminology used by authors to describe the same theoretical constructs. Grouping considered the examples and explanations given by the study authors for each domain of tinnitus-related problem (examples not reported here). Domains that could not be coded either because they were not well-defined, described multiple theoretical constructs which did not group together, or described external modulators of the tinnitus were as follows: *Ability to mask the tinnitus sound; Aggravated by noise; Auditory perceptual characteristics of tinnitus; Catastrophic; Changes for loud background noise; Changes in perception over time; Effects of tinnitus on health; Effects of tinnitus on the patients social, emotional and physical behaviour; Emotional; Emotional reaction, social activities and communication, and focused attention; Emotions; Factors that aggravate tinnitus; Functional; Functional handicap caused by tinnitus; Illness focusing; Masking effects; Medical interaction; Most problematic situation; Other people; Overall patient stress and severity of tinnitus; Psychological; Relax; Relief from tinnitus; Self-perceived tinnitus handicap; Sensations in the presence of such sounds; Situational difficulties; Situational effects; Stressors associated with onset or exacerbation of tinnitus; The extent of problems due to tinnitus; Tinnitus loudness/strength, annoyance, impact on life and severity; Tinnitus handicap; Tinnitus problem; Tinnitus sensation; Tinnitus severity; Tinnitus burden and severity*. (DOCX 18 kb)

Additional file 7: Grouping table reporting the different terminology used by authors to describe the same theoretical constructs describing complaints reported by significant others who are members of the family of a person with tinnitus. Each grouping considered the examples and/or explanations given by the study authors for each problem domain (examples reported in the text). All data come from just two studies [23, 31]. (DOCX 14 kb)

## Abbreviations

CINAHL: Cumulative Index to Nursing and Allied Health Literature
HR-QoL: Health-related quality of life questionnaire
MeSH: Medical subject headings
PICOS: Patient, Intervention, Comparison, Outcome, Setting
TEQ: Tinnitus Effects Questionnaire
THI: Tinnitus Handicap Inventory
THQ: Tinnitus Handicap Questionnaire
TQ: Tinnitus Questionnaire
TRQ: Tinnitus Reaction Questionnaire
TSI: Tinnitus Severity Index
WHO: World Health Organization
