# Supplementary material for: A narrative synthesis of research evidence for tinnitus-related complaints as reported by patients and their significant others
Source: Health Qual Life Outcomes. 2018 Apr 11;16:61. doi: 10.1186/s12955-018-0888-9 (PMC5896078; doi:10.1186/s12955-018-0888-9)
Supplement: Supplementary file 1 — Table summarising the electronic information sources used to identify the 3580 records. For a description of the abbreviations, see text. (DOCX 17 kb) [file 12955_2018_888_MOESM1_ESM.docx]

**Additional file 1.** Table summarising the electronic information sources used to identify the 3580 records. For a description of the abbreviations, see text.

| **Type of electronic information source** | **Database** | **Number of records** |
| --- | --- | --- |
| Academic literature | PubMed | 700 |
|  | Embase | 1000 |
|  | CINAHL | 26 |
| Grey literature | Open Grey | 23 |
|  | PsycEXTRA | 23 |
|  | DART | 99 |
|  | ProQuest Dissertations | 932 |
|  | Networked Digital Library | 168 |
|  | Cos Conference Papers Index | 226 |
|  | Web of Science | 257 |
| Non-specific | Google and Google Scholar | 126 |
