# Supplementary material for: A narrative synthesis of research evidence for tinnitus-related complaints as reported by patients and their significant others
Source: Health Qual Life Outcomes. 2018 Apr 11;16:61. doi: 10.1186/s12955-018-0888-9 (PMC5896078; doi:10.1186/s12955-018-0888-9)
Supplement: Supplementary file 2 — Fourteen non-English language records that were screened and either excluded or included at the full-text stage by native language speakers. ‘Complaints not reported’ = authors reported the global tinnitus score calculated from a multi-attribute questionnaire without reporting the component domains or subscales. (DOCX 18 kb) [file 12955_2018_888_MOESM2_ESM.docx]

**Additional File 2.** Fourteen non-English language records that were screened and either excluded or included at the full-text stage by native language speakers. ‘Complaints not reported’ = authors reported the global tinnitus score calculated from a multi-attribute questionnaire without reporting the component domains or subscales.

| **Reference** | **Language** | **Excluded/**  **Included** | **Reason** |
| --- | --- | --- | --- |
| Greimel KV, Leibetseder M, Unterrainer J, Albegger K. Can tinnitus be measured? Methods for assessment of tinnitus-specific disability and presentation of the Tinnitus Disability Questionnaire [Ist Tinnitus meßbar? Methoden zur Erfassung tinnitusspezifischer Beeintrachtigungen und Prasentation des Tinnitus-B.] HNO. 1999;47(3):196–201. | German | Excluded: Full-text screening | Complaints not reported |
| Hesse, S., Meyer, A., Singer, S. and Hinz, A. (2012). Mental distress and quality of life in tinnitus patients. Laryngo- Rhino- Otologie. 91 (12). p.pp. 774–781. | German | Excluded: full-text screening | Complaints not reported |
| Hubatsch L. Investigation of anxiety disorder and stress perception in chronic tinnitus [Untersuchung von Angststörung und Stressempfinden bei chronischem Tinnitus]. Charité – Universitätsmedizin Berlin. 2013. | German | Excluded: full-text screening | Complaints not reported |
| Kaluzny W, Durk, Pajor A. Impact of tinnitus on quality of life in the patients’ self-assessment [Wpływ szumóW usznych na iakość życia W samoocenie chorych]. Otolaryngologia Polish journal of Otolaryngology. 2005;59:271–76. | Polish | Included |  |
| Mao K., Jiang W, Feng, Y. Preliminary investigation of psychologic factors in 76 tinnitus patients. Journal of Clinical Otorhinolaryngology Head and Neck Surgery. 2011;25(16):732–34. | Chinese | Excluded: Full-text screening | Out of scope |
| Perrig-Chiello P, Gusset S. Tinnitus aurium: Differential aspects of the subjectively perceived strain [Differentielle Aspekte der subjektiven Belastung durch Tinnitus aurium]. Psychotherapie, Psychosomatik, Medizininische Psychologie. 1996;46:139–46. | German | Included |  |
| Schaaf H, Eipp C, Deubner R, Hesse G, Vasa R, Gieler, U. Psychosocial aspects of coping with tinnitus and psoriasis patients : A comparative study regarding suicidality, anxiety and depression [Psychosoziale Aspekte der Krankheitsverarbeitung bei Tinnitus- und Psoriasis-Patienten : Eine Vergleichsstudie hinsic. HNO. 2009;57(1):57–63. | German | Excluded: full-text screening | Tinnitus not main focus |
| Stobik C, Weber RK, Munte TF, Frommer J. Psychosomatic stress factors in compensated and decomplusated tinnitus. Psychotherapie, Psychosomatik Medizinische Psychologie. 2003;53(8):344–52. | German | Included |  |
| Stürz K, Viertler HP, Kopp M, Pfaffenberger N, Günther V. Quality of partnerships in patients with tinnitus [Die Qualität der Partnerschaft von Tinnituspatienten]. HNO. 2008;56:701–06. | German | Excluded: full-text screening | Complaints not reported |
| Urnau D, Tochetto TM. Characteristics of the tinnitus and hyperacusis in normal hearing individuals [Características do zumbido e da hiperacusia em indivíduos normo-ouvintes]. Arquivos Internacionais de Otorrinolaringologia. 2011:15(4):468–74. | Portuguese | Included |  |
| Wang H, Zhou Y, Zhai S, Yang W. Psychological aspects of subjective tinnitus. Journal of Clinical Otorhinolaryngology. 2003;17:14-5. | Mandarin Chinese | Excluded: Full-text screening | Out of scope |
| Weber SR, Périco E. Tinnitus in noise-exposed workers [Zumbido no trabalhador exposto ao ruído]. Revista da Sociedade Brasileira de Fonoaudiologia. 2011;16(4):459–65. | Portuguese | Excluded: full-text screening | Complaints not reported |
| Xu J, Zheng Y, Meng Z, Li M. The relationship between tinnitus severity and anxiety. Journal of Clinical Otorhinolaryngology Head And Neck Surgery. 2012;26(16):729–32. | Mandarin Chinese | Excluded: Full-text screening | Out of scope |
| Zirke N. Tinnitus and psychiatric comorbidtities. [Tinnitus und psychische Komorbiditäten]. Charité - Universitätsmedizin Berlin. 2014. | German | Excluded: full-text screening | Duplication (chapter with complaints reported published) |
