# Supplementary material for: A narrative synthesis of research evidence for tinnitus-related complaints as reported by patients and their significant others
Source: Health Qual Life Outcomes. 2018 Apr 11;16:61. doi: 10.1186/s12955-018-0888-9 (PMC5896078; doi:10.1186/s12955-018-0888-9)
Supplement: Supplementary file 3 — Records that were excluded because either the abstracts and/or full-texts were not accessible, or there was an incomplete reference which meant that the article could not be traced. (DOCX 35 kb) [file 12955_2018_888_MOESM3_ESM.docx]

**Additional File 3.** Records that were excluded because either the abstracts and/or full-texts were not accessible, or there was an incomplete reference which meant that the article could not be traced.

| **Excluded at the stage of abstract assessed for eligibility because the abstract was not accessible (n=39)** |
| --- |
| Ambrosino SV. Neuropsychiatric aspects of tinnitus. The Journal of Laryngology and Otology. 1981;4(Supplement):169-72. |
| Becht AC. An exploratory study: the effects of subjective tinnitus on health in deaf and hearing adults. 1982. Master's thesis, University of Oregon. 75 pages. |
| Bernabei A, Bertagna G, Cianfrone G, Spadafora R. Organization of personality in subjects with tinnitus. Organizzazione Della Personalita In Soggetti Portatori Di Acufene 1984;3:271-7. |
| Bush FM, Martelli J. Tinnitus and earache-long-term studies in 105 patients with temporomandibular (TM) disorders. Journal of Dental Research. 1986;65:185. |
| Ciocon JO, Ciocon DG. Profile of the elderly with tinnitus and effect on functional status. Journal of the American Geriatrics Society 1995;43(9):SA28. |
| Clarke L. The Noise: you too can cope with tinnitus. Web page (Caritsa). 1988;65:11-2. |
| Dauman R. Conceptual issues in the measurement of outcomes in tinnitus suffering patients. International Journal of Rehabilitation Research 2004;27:152-3. |
| Davis A, Tyler RS, Conradarmes D. Assessment of tinnitus by questionnaire and audiological methods. Clinical Otolaryngology 1980;5(6):419-420. |
| El Refaie A, Davis A, Kayan A, Baskill J, Owen V. Quality of life issues in tinnitus sufferers: Prospective study on illness perception. Psychology & Health 2008 23(Suppl 1);110-1. |
| El Refaie A, Davis A, Kayan A, Baskill J, Lovell E, Owen V. Quality of Life issues in tinnitus sufferers. Ninth International Congress of Behavioral Medicine. November 2006. |
| Folmer RL, Martin WH. Characteristics of chronic tinnitus resulting from head or neck injuries. Neurology 2001;56(8):A35-6. |
| Goebel G. Tinnitus--often a common nuisance to patients and experts. Why does troublesome tinnitus turn the afflicted person into a troublesome patient? Web page (Medizinisch-Psychosomatische Klinik Roseneck, Prien/Chiemsee). 1994 |
| Goebel G, Hiller W, Rief W, Fichter M. Behavioral medicine diagnosis in chronic tinnitus using a tinnitus inventory. Verhaltenstherapie 1995;5:A66. |
| Greimel KV, Biesinger E. Outpatient treatment concepts in chronic tinnitus. Verhaltenstherapie 1999;9:21. |
| Heinecke, K. Attention deficits in distressed tinnitus sufferers is there an objective impairment? Ninth International Congress of Behavioral Medicine November 2006. |
| Hiller W. Goebel G. The relationship between loudness and annoyance in tinnitus. Ninth International Congress of Behavioral Medicine November 2006. |
| Jakes S. Tinnitus – Psychological aspects. Bulletin of the British Psychological Society. 1986(39):A45. |
| Kasper D, Brand S, Hug JE, Holsboer-Trachsel E, Hatzinger M, Pueschel J. Outcome of Cognitive-behavioural Therapy to treat tinnitus is related to depressive symptomatology. Neuropsychobiology 2009;59(2):66-7. |
| Konzag TA, Rubler D, Fikentscher E. Interpersonal problems, disease processing and comorbidity in tinnitus outpatients. Psychotherapie Psychosomatik Medizinische Psychologie 2003;53(2):117-8. |
| Kroener-Herwig B. Do patient variables predict therapy outcome in tinnitus treatment? Ninth International Congress of Behavioral Medicine November 2006. |
| Kuyper P, Fransestekelenburg R, Dreschler W. Problems in the investigation and the treatment of tinnitus. Clinical Otolaryngology 1987;12(1):75. |
| Lamprecht F, Jaeger B. Preconditions and models of successful coping with tinnitus. Psychosomatic Medicine 1997;59(1):84. |
| Lesmann N, Schwab R, Graul J, Nutzinger DO. The significance of copying styles and social support for tinnitus patients. Verhaltenstherapie 1999;9:46. |
| Maloney G, Matignon E, Mehta N, Forgione A. SCL-90 scores and symptoms of tinnitus-TMD, TMD patients and controls. Journal of Dental Research 1995;74:222. |
| Maloney G, Matignon E, Mehta N, Forgione A, Clark RE. SCL-90 nonspecific physical symptoms in tinnitus vs non-tinnitus TMD patients. Journal of Dental Research 1996;75:2684. |
| Mattia, GM. Tinnitus: A real problem to solve. Conference of the Acoustical Society of The Netherlands. October 2001. |
| Narozny WA, Kuczkowski JE, Mikaszewski BO. Measuring severity of tinnitus with a visual analog scale. American Family Physician. 2005;71(5):855-6. |
| Ooms E, Vanheule S, Vinck B. Subjective severity in tinnitus as a difficulty in binding affective arousal. World Congress on Psychosomatic Medicine. September 2009. |
| Piera A, Lainez MJ, De Paula C, Avila C, Campos S. Chronic tinnitus: A neurological problem. Neurology 2008;70(11):A263. |
| Prytulla I, Tonnies S, Graul J, Nutzinger DO. Comparison of coping strategies in compensated and decompensated tinnitus. Verhaltenstherapie 1999;9:62. |
| Robinson SK, McQuaid JR, Viirre ES, Harris JP, Miller DL, Betzig LL. Reliability of measures of tinnitus and their association with psychological variables. Psychosomatic Medicine 2001;63(1):140-1. |
| Sanchez T. Somatosensory tinnitus: Mechanisms, clinical characteristics and management. Ninth European Federation of Audiology Societies Congress. June 2009. |
| Storz S, Novacek T, Goebel G, Hellbruck J. Coping with illness in cases of impaired hearing and tinnitus as part of inpatient behavioral therapy. Verhaltenstherapie 1999;9:75. |
| Stover B, Henry J, Zaugg T, Owens K, Kaelin C, Griest S. 2009. Tinnitus Presence Questionnaire preliminary results. Annual Meeting of the American Auditory Society. March 2009. |
| Svitak M, Rief W, Goebel G, Fichter M. Psychological characteristics of person with decompensated tinnitus. Verhaltenstherapie 1999;9:76. |
| Weise C, Hesser H, Andersson G. Tinnitus distress–Relationships with depression, catastrophizing and help-seeking. World Congress of Behavioral and Cognitive Therapies, Boston, June 2010. |
| Williams K, Tucker D. Physical activity levels in patients with tinnitus. Journal of Sport & Exercise Psychology 2005;27:S158-9. |
| Wise K, Muller W, Goebel G, Hiller W. Tinnitus patients: Coping strategies and their evolution during inpatient treatment. Verhaltenstherapie 1999; 9:81. |
| **Excluded at the stage of abstract assessed for eligibility because the reference was incomplete (n=5)** |
| B.F. Tinnitus: When it rings in the patient’s ear. Deutsche Apotheker-Zeitung. 2011;151:79. |
| Jaeger B, Malewski P, Koch T, Scwab B, Lamprecht F. Prediction of the initial adaptation to tinnitus. First results of the Hannover-Tinnitus-Longitudinal Study (HTLS). Conference paper. 2003. |
| Lamprecht F, Jaeger B. Preconditions and models of successful coping with tinnitus. 2000. Conference paper. |
| Schwab B, Lenarz T. Hearing loss and tinnitus. Springer Verlag. 2001:pp131-3 |
| Zsuzsanna, Z. Tinnitus and anxiety-depression. Is there any correlation? 6th European Congress of Oto-Rhino-Laryngology Head and Neck Surgery June 2007. |
| **Excluded at the stage of full text assessed for eligibility (electronic search) because only the abstract was available (n=11)** |
| Asnis G, Majeed K, Henderson M, De La Garza R. Tinnitus co-morbid with insomnia: A significant interrelationship and implications. International Journal of Neuropsychopharmacology 2012;15:241. |
| Freidenberg BM, Blanchard EB. Emotional and functional impairment from chronic tinnitus. Applied Psychophysiology and Biofeedback 2003;28(4);322-3. |
| Herbert S, Gagne A, Carrier J. Subjective sleep disturbances in tinnitus patients are related to their subclinical depressive symptoms. Sleep 2006;29:A315-6. |
| Jastreboff, PJ. The relative role of conscience and subconscience as related to tinnitus severity. 36th Annual Meeting of the Society for Neuroscience. October 2006. |
| Langenbach M. Psychosocial predictors of decompensation of acute tinnitus aurium. Journal of Psychosomatic Research 2002;52(5):401. |
| Moschen R, Schlatter A, Rumpold G, Schmidt A. Validation of the Chronic Tinnitus Acceptance Questionnaire (CTAQ). Journal of Psychosomatic Research 2010;68(6):650. |
| Ozcankaya, R. 2001. Depression and anxiety symptoms in tinnitus patients. Scientific Publishers of India. 3:221-4. (incomplete reference) |
| Scott B, Lindberg P. Psychological profiles in help-seeking and non-help-seeking tinnitus subjects. International Journal of Psychology. 2000;35(3-4). |
| Stege U, Joachim R, Stege K, Kischkel E, Mazurek B, Reisshauer A. Psychological comorbidity and coping strategies in patients with chronic tinnitus. International Journal of Psychology 2008;43(3-4):476. |
| Stobik C, Weber RK, Munte TF, Frommer J. Psychosomatic stress factors in compensated and decompensated tinnitus. Psychotherapie Psychosomatik Medizinische Psychologie 2004;54(2):113. |
| Zumbaum-Fischer FO, Georgiewa P, Mazurek B, Klutentreter A, Walter OB, Schmidt M, Seydel C, Klapp BF. Stress experience of patients with compensated and decompensated chronic tinnitus-a comparative study. Psychotherapie Psychosomatik Medizinische Psychologie 2005;55(2):160. |
| **Excluded at the stage of full text assessed for eligibility (manual search) because only the abstract was available (n=1)** |
| Sullivan MD, Katon W, Dobie R, Sakai C, Russo J, Harrop-Griffiths J. Disabling tinnitus. Association with affective disorder. Gen Hosp Psychiatry. 1988;10(4):285-91. |
