# Supplementary material for: A narrative synthesis of research evidence for tinnitus-related complaints as reported by patients and their significant others
Source: Health Qual Life Outcomes. 2018 Apr 11;16:61. doi: 10.1186/s12955-018-0888-9 (PMC5896078; doi:10.1186/s12955-018-0888-9)
Supplement: Supplementary file 4 — References for all 84 full-texts included for data collection and synthesis. (DOCX 22 kb) [file 12955_2018_888_MOESM4_ESM.docx]

**Additional File 4.** References for all 84 full-texts included for data collection and synthesis.

| Ahnblad P, Nordkvist A. A randomized, placebo-controlled, double-blind, parallel groups study evaluating the performance and safety of a steady state coherent biomodulator patch in the treatment of subjective tinnitus. Int Tinnitus J. 2017;21(2):157-67. |
| --- |
| Alsanosi AA. Impact of tinnitus on quality of life among Saudi patients. Saudi Med J. 2011;32(12):1274–8. |
| Andersson G, Lyttkens L, Larsen HC. Distinguishing levels of tinnitus distress. Clin Otolaryngol. 1999;24:404–10. |
| Andersson G, Strömgren T, Ström L, Lyttkens L. Randomized controlled trial of Internet-based cognitive behaviour therapy for distress associated with tinnitus. Psychosom Med. 2002;64(5):810–6. |
| Andersson G, Edvinsson E. Mixed feelings about living with tinnitus: A qualitative study. J Audiol Med. 2008;6(1):48–54. |
| Andersson G, Porsaeus D, Wiklund M, Kaldo V, Larsen HC. Treatment of tinnitus in the elderly: A controlled trial of cognitive behavior therapy. Int J Audiol. 2005;44(11):671–5. |
| Andersson G, Vretblad P, Larsen HC, Lyttkens L. Longitudinal follow-up of tinnitus complaints. Arch Otorhinolaryngol - Head Neck Surg. 2001;127:175–9. |
| Bankstahl US, Gortelmeyer R. Measuring subjective complaints of attention and performance failures - development and psychometric validation in tinnitus of the self-assessment scale APSA. Heal Qual Life Outcomes. 2013;11(1):86. |
| Bayar N, Oguzturk O, Koc C. Minnesota Multiphasic Personality Inventory Profile of patients with subjective tinnitus. J Otolaryngol. 2002;31:317–22. |
| Berberian AP, Ribas A, Imlau D, Guarinello AC, Massi G, Tonocchi R, Riesemberg R, Martins J, Rosa MRD. Benefit of using the prosthesis with sound generators in individuals with tinnitus associated with mild to moderately severe hearing loss 2016;20(2):64-6. |
| Cabral A, Tonocchi R, Ribas A, Almeida G, Rosa M, Massi G, Berberian AP. The efficacy of hearing aids for emotional and auditory tinnitus issues. Int Tinnitus J. 2016; 20(1);54-8. |
| Cho CG, Chi JH, Song JJ, Lee EK, Kim BH. Evaluation of anxiety and depressive levels in tinnitus patients. Korean J Audiol. 2013;17:83–9. |
| Crocetti A, Forti S, Ambrosetti U, Bo L Del. Questionnaires to evaluate anxiety and depressive levels in tinnitus patients. Otolaryngol - Head Neck Surg. 2009;140(3):403–5. |
| Davis PB, Paki B, Hanley PJ. Neuromonics tinnitus treatment: Third clinical trial. Ear Hear. 2007;28(2):242–59. |
| Dineen R, Doyle J, Bench J. Audiological and psychological characteristics of a group of tinnitus sufferers, prior to tinnitus management training. Br J Audiol. 1997;31(1):27–38. |
| Drexler D, López-Paullier M, Rodio S, González M, Geisinger D, Pedemonte M. Impact of reduction of tinnitus intensity on patients' quality of life. Int J  Audiol. 2016;55(1):11-9. |
| El Refaie A, Davis A, Baskill J, Lovell E, Owen V. A questionnaire study of the quality of life and quality of family life of individuals complaining of tinnitus pre- and post- attendance at a tinnitus clinic. Int J Audiol. 2004;43(7):410–6. |
| Erlandsson SI, Hallberg LRM, Axelsson A. Psychological and audiological correlates of perceived tinnitus severity. Audiology. 1992;31:168–79. |
| Fetoni AR, Lucidi D, De Corso E, Fiorita A, Conti G, Paludetti G. Relationship between subjective tinnitus perception and psychiatric discomfort. Int Tinnitus J. 2016;20(2):76-82. |
| Ferreira LMDBM, Ramos AN, Mendes EP. Characterization of tinnitus in the elderly and its possible related disorders. Braz J Otorhinolaryngol. 2009;75(2):249–55. |
| Gomaa MAM, Elmagd MHA, Elbadry MM, Kader RMA. Depression, anxiety and stress scale in patients with tinnitus and hearing loss. Eur Arch Oto-Rhino-Laryngology. 2014;271(8):2177–84. |
| Granjeiro RC, Kehrle HM, de Oliveira TSC, Sampaio ALL, de Oliveira CACP. Is the degree of discomfort caused by tinnitus in normal-hearing individuals correlated with psychiatric disorders? Otolaryngol Neck Surg. 2013;148(4):658–63. |
| Granqvist P, Lantto S, Ortiz L, Andersson G. Adult attachment, perceived family support, and problems experienced by tinnitus patients. Psychol Health. 2001;16(3):357–66. |
| Hallam RS. Manual of the Tinnitus Questionnaire (TQ). London: The Psychological Corporation; 1996. |
| Hallam RS, Jakes SC, Hinchcliffe R. Cognitive variables in tinnitus annoyance. Br J Clin Psychol. 1988;27:213–22. |
| Hallberg LR, Erlandsson SI. Tinnitus characteristics in tinnitus complainers and noncomplainers. Br J Audiol. 1993;27(1):19–27. |
| Handscomb LE, Hall DA, Shorter G, Hoare DJ. Positive and negative thinking in tinnitus: Factor structure of the Tinnitus Cognitions Questionnaire. Ear Hear. 2017;38(1):126-32. |
| Haralambous G, Wilson PH, Platt-Hepworth S, Tonkin JP, Hensley VR, Kavanagh D. EMG Biofeedback in the treatment of tinnitus an experimental evaluation. Behav Res Ther. 1987;25(1):49–56. |
| Harrop-Griffiths J, Katon W, Dobie R, Sakai C, Russo J. Chronic tinnitus: association with pychiatric diagnoses. J Psychosom Res. 1987;31(5):613–21. |
| Hébert S, Fullum S, Carrier J. Polysomnographic and quantitative electroencephalographic correlates of subjective sleep complaints in chronic tinnitus. J Sleep Res. 2011;20(1 Part I):38–44. |
| Hébert S, Carrier J. Sleep complaints in elderly tinnitus patients. Ear Hear. 2007;28(5):649–55. |
| Henry JA, Thielman EJ, Zaugg TL, Kaelin C, Schmidt CJ, Griest S, McMillan GP, Myers P, Rivera I, Baldwin R, Carlson K. Randomized controlled trial in clinical settings to evaluate effectiveness of coping skills education used with Progressive Tinnitus Management. J Sp Lang Hear Res, 2017;60:1378-97. |
| Henry J, Schechter M, Zaugg T, Griest S, Jastreboff P, Vernon J, et al. Outcomes of clinical trial: Tinnitus masking versus tinnitus retraining therapy. J Am Acad Audiol. 2006;17(2):104–32. |
| Henry JL, Kangas M, Wilson PH. Development of the psychological impact of tinnitus interview: A clinician-administered measure of tinnitus-related distress. Int Tinnitus J. 2001;7(1):20–6. |
| Hiller W, Goebel G. A psychometric study of complaints in chronic tinnitus. J Psychosom Res. 1992;36(4):337–48. |
| Hiller W, Goebel G. Assessing audiology, pathophysiological, and psychological variables in chronic tinnitus: A study of reliability and search for prognostic factors. Int J Behav Med. 1999;6(4):312–30. |
| Hoekstra CEL, Wesdorp FM, van Zanten GA. Socio-demographic, health, and tinnitus related variables affecting tinnitus severity. Ear Hear. 2014;35(5):544–54. |
| Jackson JG. The relationship between tinnitus, cognitive performance demands and on the individual. University of Hull; 2005. |
| Jakes SC, Hallam RS, Rachman S, Hinchcliffe R. The effects of reassurance, relaxation training and distraction on chronic tinnitus sufferers. Behav Res Ther. 1986;24(5):497–507. |
| Jakes S, Hallam R, Chambers C, Hinchcliffe R. A factor analytical study of tinnitus complaint behaviour. Audiology. 1985;24:195–206. |
| Kaldo V, Cars S, Rahnert M, Larsen HC, Andersson G. Use of a self-help book with weekly therapist contact to reduce tinnitus distress: A randomized controlled trial. J Psychosom Res. 2007;63(2):195–202. |
| Kaluzny W, Durko T, Pajor A. Impact of tinnitus on quality of life in the patients’ self-assessment. Polish Otolaryngol. 2005;59(2):271–6. |
| Karatas E, Deniz M. The comparison of acoustic and psychic parameters of subjective tinnitus. Eur Arch Oto-Rhino-Laryngology. 2012;269(2):441–7. |
| Kennedy V, Chéry-Croze S, Stephens D, Kramer S, Thai-Van H, Collet L. Development of the International Tinnitus Inventory (ITI): A patient-directed problem questionnaire. J Audiol Med. 2005;3(4):228–37. |
| Kleinstäuber M, Jasper K, Schweda I, Hiller W, Andersson G, Weise C. The role of fear-avoidance cognitions and behaviours in patients with chronic tinnitus. Cogn Behav Ther. 2013;42(2):84–99. |
| Kuk FK, Tyler RS, Russell D, Jordan H. The psychometric properties of a Tinnitus Handicap Questionnaire. Amplif Aural Rehabil. 1990;11(6):434–45. |
| Maes IH. Tinnitus: Assessment of quality of life and costs. Universiteit Maastricht; 2014. |
| Marciano E, Carrabba L, Giannini P, Sementina C, Verde P, Bruno C, et al. Psychiatric comorbidity in a population of outpatients affected by tinnitus. Int J Audiol. 2003;42:4–9. |
| McMullen AF. Investigation of the perception of tinnitus severity tinnitus loudness and individual locus of control. University of Cincinnati; 2001. |
| Meikle MB, Henry J a, Griest SE, Stewart BJ, Abrams HB, McArdle R, et al. The Tinnitus Functional Index: Development of a new clinical measure for chronic, intrusive tinnitus. Ear Hear. 2012;33(2):153–76. |
| Meng Z, Chen Z, Xu K, Li G, Tao Y, Kwong JS. Psychometric properties of a  Mandarin version of the tinnitus questionnaire. Int J Audiol. 2016;55(6):366-74 |
| Miguel GS, Yaremchuk K, Roth T, Peterson E. The effect of insomnia on tinnitus. Ann Otol Rhinol Laryngol. 2014;123(10):696–700. |
| Mollasadeghi A, Mirmohammadi SJ, Mehrparvar AH, Davari MH, Shokouh P, Mostaghaci M, et al. Efficacy of low-level laser therapy in the management of tinnitus due to noise-induced hearing loss: A double-blind randomized clinical trial. Sci World J. 2013;2013:1–7. |
| Newman CW, Wharton JA, Shivapuja BG, Jacobson GP. Relationships among psychoacoustic judgments, speech understanding ability and self-perceived handicap in tinnitus subjects. Audiology. 1994;33(1):47–60. |
| Newman CW, Jacobson GP, Spitzer JB. Development of the Tinnitus Handicap Inventory. 1996;122:143–8. |
| Ooms E, Vanheule S, Meganck R, Vinck B, Watelet JB, Dhooge I. Tinnitus severity and its association with cognitive and somatic anxiety: A critical study. Eur Arch Oto-Rhino-Laryngology. 2012;269:2327–33. |
| Oron Y, Sergeeva NV, Kazlak M, Barbalat I, Spevak S, Lopatin AS, Roth Y. A  Russian adaptation of the Tinnitus Handicap Inventory. Int J Audiol. 2015;54(7):485-9. |
| Koc AO, Ertugay CK, Bar SSKİ, Erbek S. Psychotic measurements of patients who presented to our clinic with subjective tinnitus symptoms. KBB-Forum. 2015;14(4):76–81. |
| Perrig-Chiello P, Gusset S. Differentielle aspekte der subjektiven belastung durch tinnitus aurium [Tinnitus aurium: Differential aspects of the subjectively perceived strain]. Psychother Psychosom Medizininische Psychol. 1996;46:139–46. |
| Rief W, Weise C, Kley N, Martin A. Psychophysiologic treatment of chronic tinnitus: A randomized clinical trial. Psychosom Med. 2005;67(5):833–8. |
| Rutter DR, Stein MJ. Psychological aspects of tinnitus: A comparison with hearing loss and ear, nose and throat disorders. Psychol Heal. 1999;14(4):711–8. |
| Sadlier M, Stephens SDG, Kennedy V. Tinnitus rehabilitation: A mindfulness meditation cognitive behavioural therapy approach. J Laryngol Otol. 2008;122(1):31–7. |
| Sahlsten H, Isohanni J, Haapaniemi J, Salonen J, Paavola J, Löyttyniemi E,  Johansson R, Jääskeläinen SK. Electric field navigated transcranial magnetic  stimulation for chronic tinnitus: A pilot study. Int J Audiol. 2015;54(12):899-909. |
| Sanchez L, Stephens SDG. Perceived problems of tinnitus clinic clients at long-term follow up. J Audiol Med. 2000;9(2):94–103. |
| Sanchez L, Stephens SDG. A tinnitus problem questionnaire in a clinic population. Ear Hear. 1997;18(3):210–7. |
| Schaaf H, Weiß S, Hesse G. Catamnesis results of an inpatient neuro-otologic and psychosomatic tinnitus therapy 1-5 years after discharge. Eur Arch Otorhinolaryngol. 2017;274(2):701-10. |
| Scott B, Lindberg P. Psychological profile and somatic complaints between help-seeking and non-help-seeking tinnitus subjects. Psychosomatics. 2000;41(4):347–52. |
| Seydel C, Haupt H, Olze H, Szczepek AJ, Mazurek B. Gender and chronic tinnitus: Differences in tinnitus-related distress depend on age and duration of tinnitus. Ear Hear. 2013;34(5):661–72. |
| Smith SL, Fagelson M. Development of the self-efficacy for tinnitus management questionnaire. J Am Acad Audiol. 2011;22:424–40. |
| Sourgen PM, Ross E. Perceptions of tinnitus in a group of senior citizens. South African J Commun Disord. 1998;45:61–75. |
| Stiegler P, Matzi V, Lipp C, Kontaxis A, Klemen H, Walch C, et al. Hyperbaric oxygen (HBO2) in tinnitus: Influence of psychological factors on treatment results? J Undersea Hyperb Med Soc. 2006;33(6):429–37. |
| Stobik C, Weber RK, Munte TF, Frommer J. Psychosomatic stress factors in compensated and decompensated tinnitus. Psychother Psychosom Medizinische Psychol. 2003;53(8):344–52. |
| Stobik C, Weber RK, Münte TF, Walter M, Frommer J. Evidence of psychosomatic influences in compensated and decompensated tinnitus. Int J Audiol. 2005;44(6):370–8. |
| Toft S. Tinnitus : Effects on the patient and partner. University of Leicester; 2003. |
| Tyler RS, Haihong J, Perreau A, Witt S, Noble W, Coelho C. Development and validation of the Tinnitus Primary Function Questionnaire. Am J Audiol. 2014;23:260–72. |
| Tyler RS, Noble W, Coelho CB, Ji H. Tinnitus retraining therapy: Mixing point and total masking are equally effective. Ear Hear. 2012;33(5):588–94. |
| Tyler RS, Baker LJ. Difficulties experienced by tinnitus sufferers. J Speech Hear Disord. 1983;48(2):150–4. |
| Urnau D, Tochetto TM. Characteristics of the tinnitus and hyperacusis in normal hearing individuals [Características do zumbido e da hiperacusia em indivíduos normo-ouvintes]. Arq Int Otorrinolaringol. 2011;15(4):468–74. |
| Vanneste S, Figueiredo R, De Ridder D. Treatment of tinnitus with cyclobenzaprine: An open-label study. Int J Clin Pharmacol Ther. 2012;50(5):336–44. |
| Wagenaar OV, Wieringa M, Mantingh L, Kramer SE, Kok R. Preliminary longitudinal results of neuropsychological education as first and sole intervention for new tinnitus patients. Int Tinnitus J. 2016 22;20(1):11-7. |
| Wakabayashi S, Saito H, Oishi N, Shinden S, Ogawa K. Effects of tinnitus treatments on sleep disorders in patients with tinnitus. Int J Audiol. 2017;14:1-5. |
| Wallhäusser-Franke E, Brade J, Balkenhol T, D’Amelio R, Seegmüller A, Delb W. Tinnitus: Distinguishing between subjectively perceived loudness and tinnitus-related distress. PLoS One. 2012;7(4):1–7. |
| Wilson PH, Henry J, Bowen M, Haralambous G. Tinnitus Reaction Questionnaire: Psychometric properties of a measure of distress associated with tinnitus. J Speech Hear Res. 1991;34(1):197–201. |
| Zirke N, Seydel C, Szczepek AJ, Olze H, Haupt H, Mazurek B. Psychological comorbidity in patients with chronic tinnitus: Analysis and comparison with chronic pain, asthma or atopic dermatitis patients. Qual Life Res. 2013;22(2):263–72. |
