# Supplementary material for: A narrative synthesis of research evidence for tinnitus-related complaints as reported by patients and their significant others
Source: Health Qual Life Outcomes. 2018 Apr 11;16:61. doi: 10.1186/s12955-018-0888-9 (PMC5896078; doi:10.1186/s12955-018-0888-9)
Supplement: Supplementary file 6 — Grouping table reporting the different terminology used by authors to describe the same theoretical constructs. Grouping considered the examples and explanations given by the study authors for each domain of tinnitus-related problem (examples not reported here). Domains that could not be coded either because they were not well-defined, described multiple theoretical constructs which did not group together, or described external modulators of the tinnitus were as follows: Ability to mask the tinnitus sound; Aggravated by noise; Auditory perceptual characteristics of tinnitus; Catastrophic; Changes for loud background noise; Changes in perception over time; Effects of tinnitus on health; Effects of tinnitus on the patients social, emotional and physical behaviour; Emotional; Emotional reaction, social activities and communication, and focused attention; Emotions; Factors that aggravate tinnitus; Functional; Functional handicap caused by tinnitus; Illness focusing; Masking effects; Medical interaction; Most problematic situation; Other people; Overall patient stress and severity of tinnitus; Psychological; Relax; Relief from tinnitus; Self-perceived tinnitus handicap; Sensations in the presence of such sounds; Situational difficulties; Situational effects; Stressors associated with onset or exacerbation of tinnitus; The extent of problems due to tinnitus; Tinnitus loudness/strength, annoyance, impact on life and severity; Tinnitus handicap; Tinnitus problem; Tinnitus sensation; Tinnitus severity; Tinnitus burden and severity. (DOCX 18 kb) [file 12955_2018_888_MOESM6_ESM.docx]

**Additional File 6*.*** Grouping table reporting the different terminology used by authors to describe the same theoretical constructs reported by patients. Grouping considered the examples and explanations given by the study authors for each domain of tinnitus-related problem (examples not reported here). Domains that could not be coded either because they were not well-defined, described multiple theoretical constructs which did not group together, or described external modulators of the tinnitus were as follows: *Ability to mask the tinnitus sound; Aggravated by noise; Auditory perceptual characteristics of tinnitus; Catastrophic; Changes for loud background noise; Changes in perception over time; Effects of tinnitus on health; Effects of tinnitus on the patients social, emotional and physical behaviour; Emotional; Emotional reaction, social activities and communication, and focused attention; Emotions; Factors that aggravate tinnitus; Functional; Functional handicap caused by tinnitus; Illness focusing; Masking effects; Medical interaction; Most problematic situation; Other people; Overall patient stress and severity of tinnitus; Psychological; Relax; Relief from tinnitus; Self-perceived tinnitus handicap; Sensations in the presence of such sounds; Situational difficulties; Situational effects; Stressors associated with onset or exacerbation of tinnitus; The extent of problems due to tinnitus; Tinnitus loudness/strength, annoyance, impact on life and severity; Tinnitus handicap; Tinnitus problem; Tinnitus sensation; Tinnitus severity; Tinnitus burden and severity.*

| **Our chosen domain terminology** | **Other terminology used by Authors** |
| --- | --- |
| Ability to ignore | Capacity to ignore tinnitus; Self-attention to the tinnitus; Ability to divert attention from their tinnitus; Ignorability; Routine Tinnitus Management |
| Acceptance of tinnitus | Tolerance |
| Anger | N/A |
| Annoyance | Annoyance caused by tinnitus; Annoyance of tinnitus; Tinnitus annoyance; Annoyance/discomfort; Annoying; Disturbed by tinnitus; Permanent annoyance; Sense of annoyance generated by the tinnitus; Discomfort of tinnitus; Annoyance, irritation, inability to relax |
| Anxiety | Anxious depression; Anxiety and depression; Cognitive anxiety; Feeling anxious; Psychological comorbidity; Severity of anxiety and depressive complaints; Tension; Generalized anxiety symptoms |
| Bodily complaints | Somatic complaints; Somatic symptoms; Somatic symptom severity; Psychological-Tension; Psychosomatic; Chronic comorbidities; Pain/headaches |
| Change in sense of self | Feeling insecure; Psychological-Self image; Tinnitus identity-Comparative; Tinnitus identity-Private identity |
| Cognitive difficulties | Cognitive; Cognitive distress; Cognitive disturbance; Cognitive effects; Cognitive failures and mishaps; Cognitive stress; Dysfunctional cognition; Cognitive interference; Problems with prospective everyday memory including capacity-consuming monitoring |
| Concentration difficulties | Concentration; Concentration problems; Interference with concentration; Impairment of concentration; Inability to concentrate; Confusion; Attention and performance deficits; Attention deficit; Effects of tinnitus on mental activities; Emotional status; Distractibility; Problems keeping attention focused, necessary to execute tasks in specific situations; Influence of background sounds or noises |
| Depression | N/A |
| Depressive symptoms | Depressitivity; Depressive reactions; Generalized depressive symptoms; Depression; Psychological-Depression/low mood |
| Difficulties coping | Avoidance of situations because of tinnitus; Tinnitus-related fear avoidance behavior; Avoidant-oriented coping; Avoid noisy situation; Avoid quiet situation; Emotion-oriented coping; Cognitive and behavioral coping strategies in response to tinnitus; Cope with tinnitus; Coping; Ear-related fear avoidance behavior; Proactive coping; Self-efficacy; Sense of coherence (comprehensibility, manageability and meaningfulness); Tinnitus identity-Public identity; Quality of life; Use of auricular protection; Requiring noisy situations; Requiring quiet situations |
| Difficulties relaxing | Disturbance of relaxation; General level of relaxation; Interference with rest and relaxation; Relax; Relaxed; Relaxation |
| Distress from bodily complaints | Fear of anxiety-related somatic sensations; Psychological and psychosomatic distress; Somatisation; Somatoformic disturbance |
| Fear | Fear; Fear- avoidance cognitions; Fear of arousal-related sensations and their consequences |
| Feeling tired | Energetic; Tired; Tiredness |
| Frustration | Need speech repeated; Social-Ignorance/lack of knowledge |
| Functional difficulties due to tinnitus | Functional; Functional limitations |
| General distress | Emotional complaints; Negative affect; Perceived stress; Satisfied; Stress |
| Health-related quality of life | Quality of life; Daily life activities; Effect on life; General well-being; Impact on life; Overall enjoyment of life; Expectancies-Lifestyle/psychological factors |
| Irrational beliefs | N/A |
| Irritable | Irritability due to tinnitus; Irritation of perceived stress from daily-life events; Emotional status |
| Lack of joy | Loss of joy; Feeling cheerful |
| Lack of support from family and friends | Contact with family and friends; Others' reactions to the tinnitus sufferer; Patients views of tinnitus; Perceived attitudes; Social support; Tinnitus identity-Public identity |
| Loss of appetite | Appetite |
| Loss of control | Control over tinnitus; Controllability; Reduced sense of control; Sense of control |
| Loss of peace | Peace of mind |
| Mood states | Mood; Subjective mood; Reaction to stress |
| Need for knowledge | Patient’s personal viewpoint of tinnitus; Positive-Experience of knowing about tinnitus; Explaining tinnitus to others |
| Negative effects on hearing (tinnitus) | Hearing; Hearing difficulties; Hearing difficulty; Hearing problems; Hearing difficulty related to tinnitus; Perceived effect of tinnitus on hearing; Interference with hearing; Auditory; Auditory perceptual difficulties; Auditory perceptual difficulties attributable to tinnitus; Auditory problems; Auditory-Sound distortion; Distortion of sounds; Effects of tinnitus on hearing; Difficulty understanding speech in noise; Emotional status; Interference with passive auditory entertainment; Internal thoughts and interaction with others; Patients' hearing ability; Understanding television; Understanding speech; Listening to radio; Appreciation of music; Use of telephone; Localization of sounds; Listening to environmental sounds (doorbell, telephone bell) |
| Negative impact on activities | Avoidance of or interference with activities; Interference with work and leisure activities; Activities; Avoidance of situations because of tinnitus; Refrain from activities; Quality of life; Positive-Changed view of the world |
| Negative impact on individual activities | Interference with daily activities; Learning to drive |
| Negative impact on social life | Social-Social life affected; Social distress or impact; Effects of tinnitus on social activities; Leisure; Leisure interference; Avoidance of activities; Withdraw, avoid friends |
| Negative impact on work activities | Effects of tinnitus on work activities; Interferes with work; Social-Work; Work interference; Occupational impairments |
| Negative impact on relationships | Social-How other people are affected; Marital quality; Marital difficulty; Insecurity of attachment (ambivalence); Insecurity of attachment (avoidance); Family problems |
| Negative thoughts about tinnitus | Negative cognitions; Pessimism; Catastrophic |
| Pain | Pain complaints |
| Physical health problems | Effects of tinnitus on general health; Effects on general health/healthcare; Health; Health problems; Physical effects of tinnitus; Psychological-Lack of energy; Sick; General ill-health; Somatic and physical complaints due to tinnitus; Dependence on drugs; Ineffectiveness of drugs; Giddiness/imbalance/fuzzy head; |
| Quality of life associated with tinnitus | Tinnitus-related quality of life; Changes in life due to tinnitus; Effects of tinnitus on quality of life; Effects of tinnitus on life-style; Social handicap-The impact of tinnitus on daily living; Tinnitus-related disturbance (lifestyle disturbance); Effects on lifestyle; Enjoyment of life; Global impact on life/quality of life; Individual’s physical health, social, emotional; Disability/handicap; Effect of tinnitus on daily lives/repercussion on the lives of elderly patients; Health; Impact of tinnitus on daily life; Impact of tinnitus on daily living; Impact of tinnitus on daily life/handicap; Negative impact on quality of life; Impaired quality of life; Psychosocial impairments |
| Reduced sound tolerance | Auditory-Hyperacusis; Sounds considered uncomfortable |
| Sexual difficulties | Sexual problems |
| Sleep difficulties | Auditory-Disturbs sleep; Difficulties experienced related to sleep; Disturbance of sleep and rest; Fall asleep; Influence on sleep; Insomnia caused by tinnitus; Night’s sleep; Problems getting to sleep; Problems with relaxation and sleep; Psychological-Insomnia; Quality of sleep; Sleep; Sleep disorders; Sleep disturbance; Sleep disturbance (insomnia); Sleep efficiency; Sleep impairment; Sleep interference; Sleep problems; Sleep quality; Sleeps difficulties; Waking during night |
| Suicidal thoughts | Suicidal risk; Suicidal thoughts and actions; Considered suicide; Tinnitus-related distress |
| Tinnitus awareness | Triggers to tinnitus; Awareness; Awareness - most aware when; Awareness during the day; Awareness of tinnitus; Constant awareness; Notice tinnitus; Presence or awareness of tinnitus; Tinnitus persistence; Tinnitus obtrusiveness |
| Tinnitus intrusiveness | Intrusive; Intrusiveness; Intrusiveness of tinnitus; Unpleasantness, intrusiveness |
| Tinnitus location | Localization of tinnitus |
| Tinnitus loudness | Intensity of tinnitus; Loudness; Loudness of tinnitus; Perceived tinnitus loudness; Tinnitus loudness perception; Severity of tinnitus; Tinnitus distress; Tinnitus intensity; Effect for tinnitus intensity |
| Tinnitus pitch | N/A |
| Tinnitus-related distress | Distress attributed to tinnitus; Severe signs of distress; Distress caused by tinnitus; Severity of distress caused by tinnitus; Tinnitus distress; Tinnitus stress; Despair, frustration, depression; Tinnitus annoyance and distress; Cognitive and emotional distress; Emotional and cognitive distress; Emotional distress; Effects of tinnitus on the emotion of the sufferer; Emotional; Emotional consequences of tinnitus; Emotional disturbance; Emotional effects; Emotional effects of tinnitus; Emotional problems; Emotional reaction to tinnitus; Emotional response; Emotional stress; General distress; Demands; Mental disturbance; Most problematic situation; Overall effect of tinnitus; Psychological; Psychological consequences; Psychological distress; Psychological disturbance from tinnitus; Psychological problems; Reaction to tinnitus |
| Tinnitus unpleasantness | Unpleasant |
| Worries/concerns | Cognitive concerns; Physical concerns; Psychological-Worry about the future; Emotional distress; Insecurity, fear, worry; Social concerns; Treatment experiences-Ambivalence regarding knowledge |
