# Supplementary material for: A narrative synthesis of research evidence for tinnitus-related complaints as reported by patients and their significant others
Source: Health Qual Life Outcomes. 2018 Apr 11;16:61. doi: 10.1186/s12955-018-0888-9 (PMC5896078; doi:10.1186/s12955-018-0888-9)
Supplement: Supplementary file 7 — Grouping table reporting the different terminology used by authors to describe the same theoretical constructs describing complaints reported by significant others who are members of the family of a person with tinnitus. Each grouping considered the examples and/or explanations given by the study authors for each problem domain (examples reported in the text). All data come from just two studies [23, 31]. (DOCX 14 kb) [file 12955_2018_888_MOESM7_ESM.docx]

**Additional file 7*.*** Grouping table reporting the different terminology used by authors to describe the same theoretical constructs describing complaints reported by significant others who are members of the family of a person with tinnitus. Each grouping considered the examples and/or explanations given by the study authors for each problem domain (examples reported in the text). All data come from just two studies [27; 34].

| **Our domain terminology** | **Authors’ wording** | **Author given examples** |
| --- | --- | --- |
| Difficulties coping | Coping with day-to-day activities | None reported |
| General distress | Emotional health | Upset patient has tinnitus and can’t ease it for them; Feel stressed sometimes; Patient temperamental; Get love/attention elsewhere |
| Mood states | Mood | Disturbed |
| Negative effects on hearing | Communication | Need to speak clearly; Need for repetition; Mishears some words, can be annoying; Patient doesn’t listen; Patient shouts to communicate |
| Negative impact on relationships | Effects of tinnitus on the family; Relationship; Changes in family life | Affects patient’s mood (selfish, moan, shout, irritable etc); Patient’s mood makes partner uptight; Stress including family member in an activity; Family worry; Disturbs sleep |
| Negative impact on activities | Day-to-day activities with the tinnitus sufferer; Restrictions of activities | None reported |
| Negative impact on social life | Social life | Reluctant to go out in crowds/busy places; Conversation difficult in company; Some people avoid patient because of it |
| Need for knowledge | Understanding of tinnitus | Understanding of tinnitus and its dangers |
| Physical health problems | Physical health | Partner has to do more tasks, so tired |
| Sleep difficulties | Sleep pattern | Disturbed |
